# Dependences of Magnetic Properties on the Grain Size and Hard/Soft Magnetic Phase Volume Ratio for Ce_2_Fe_14_B/α-Fe and Nd_2_Fe_14_B/α-Fe Nanocomposites

**DOI:** 10.3390/ma16155260

**Published:** 2023-07-26

**Authors:** Xiangyi Liu, Bang Zhou, Bin Yuan, Zhongwu Liu

**Affiliations:** School of Materials Science and Engineering, South China University of Technology, Guangzhou 510640, China; msxyliu@mail.scut.edu.cn (X.L.); 18846321367@163.com (B.Z.); apsheng@scut.edu.cn (B.Y.)

**Keywords:** micromagnetic simulation, nanocomposite permanent magnet, Ce_2_Fe_14_B, Nd_2_Fe_14_B, demagnetization energy

## Abstract

The magnetic properties of magnetic nanocomposites consisting of hard and soft magnetic phases are dependent not only on the intrinsic properties but also on the grain structure and volume ratio of the two phases. In this study, we performed a systematic micromagnetic simulation on the magnetic properties of Ce_2_Fe_14_B/α-Fe and Nd_2_Fe_14_B/α-Fe nanocomposites. The volume fractions of the hard magnetic Nd_2_Fe_14_B or Ce_2_Fe_14_B phase were varied from 80% to 40%, and the grain sizes of the hard magnetic phase and the soft magnetic α-Fe phase were changed independently from 10 nm to 40 nm. The results show that when the grain size of both hard and soft phases is 10 nm and the volume fraction of the hard phase is 70%, the highest maximum magnetic energy product can be obtained in both Ce_2_Fe_14_B/α-Fe and Nd_2_Fe_14_B/α-Fe nanocomposites. The hard magnetic properties of Ce_2_Fe_14_B/α-Fe nanocomposite decrease significantly when the volume fraction of the α-Fe phase exceeds 30%. However, for the Nd_2_Fe_14_B/α-Fe system, this situation only occurs when the α-Fe volume fraction exceeds 40%. The reason for this is not only because of the low anisotropic field and the smaller exchange coupling length between the soft and hard magnetic phases, but also because of the lower saturation magnetization of the hard phase. The grain size has greater effects on the magnetic properties compared to the volume fraction of the hard magnetic phase. The main reason is that as the grain size increases, the remanence of the nanocomposite decreases sharply, which also leads to a rapid decrease in the maximum magnetic energy product. The simulation results on the effects of phase ratio and grain size have been verified by experiments on melt-spun Ce_2_Fe_14_B/α-Fe alloys with various compositions prepared by melt-spinning followed by annealing for various lengths of time. Due to the influence of demagnetization energy, the hard magnetic phase with high saturation magnetization is preferred for the preparation of high-performance nanocomposite magnets.

## 1. Introduction

Rare earth–iron–boron (RE-Fe-B)-based nanocomposites have been considered a new type of permanent magnets with high performance and relatively low cost [1,2] since Coehoorn proposed the hard magnetic and soft magnetic grain structure in 1989 [3]. In 1991, Kneller and Hawig [4] proved theoretically that the exchange coupling between soft magnetic grains and hard magnetic grains leads to the hard magnetic behavior of the nanocomposites. In 1993, Skomski and Coey [5] pointed out that the theoretical magnetic energy product of anisotropic nanocomposite materials of Sm_2_Fe_17_N_3_/Fe_65_Co_35_ can reach 1 MJ/m^3^, which is significantly higher than that of sintered Nd-Fe-B magnets. Therefore, nanocomposite permanent magnets still have a lot of room for development, even isotropic nanocomposites, due to so-called remanence enhancement.

In RE-Fe-B nanocomposite alloys, the RE_2_Fe_14_B phase with a high anisotropy field serves as the hard magnetic phase, and α-Fe and/or Fe_3_B phases with high saturation magnetization serve as the soft magnetic phase. The hard magnetic phase provides high coercivity, and the soft magnetic phase gives high remanence for the nanocomposites. At the same time, the performance of nanocomposite permanent magnets is affected by the microstructure of magnets, such as grain size and grain distribution. The appropriate arrangement of hard and soft magnetic phases is important for the preparation of high-performance nanocomposite permanent magnets. Micromagnetism is recognized as one of the powerful tools to simulate the influence of the microstructure of magnets on magnetic properties. Previously, researchers studied Nd_2_Fe_14_B/α-Fe nanocomposites by micromagnetic simulation [6,7,8], and they believed that a smaller grain size results in greater remanence, while coercivity showed the opposite trend. The highest maximum magnetic energy product (BH)_max_ of Nd_2_Fe_14_B/α-Fe nanocomposites can be obtained when the volume fraction of the hard magnetic phase is 70% and the grain size is 10 nm.

Along with the microstructure, the intrinsic properties of hard and soft phases also have decisive effects on the performance of nanocomposites. However, until now, most previous micromagnetic simulations on nanocomposites were mainly based on the Nd_2_Fe_14_B/α-Fe system. The difference between other nanocomposite systems and Nd_2_Fe_14_B/α-Fe nanocomposites has not been understood. A systematic investigation of the effects of the intrinsic magnetic properties of hard magnetic phases on the properties of nanocomposites has not yet been carried out. Therefore, this work aims to clarify the fundamental behavior and optimized structure of nanocomposites with various hard magnetic phases. Due to the relatively low price of Ce_2_Fe_14_B-type permanent magnets and the significantly different intrinsic magnetic properties of Ce_2_Fe_14_B and Nd_2_Fe_14_B phases, micromagnetic simulations on Ce_2_Fe_14_B/α-Fe and Nd_2_Fe_14_B/α-Fe nanocomposites were carried out. The optimized structures for these two systems are discussed here, and reasonable suggestions for the preparation of other nanocomposite permanent magnets are provided.

## 2. Simulation and Experimental Methods

### 2.1. Simulation Method

**Figure 1:**
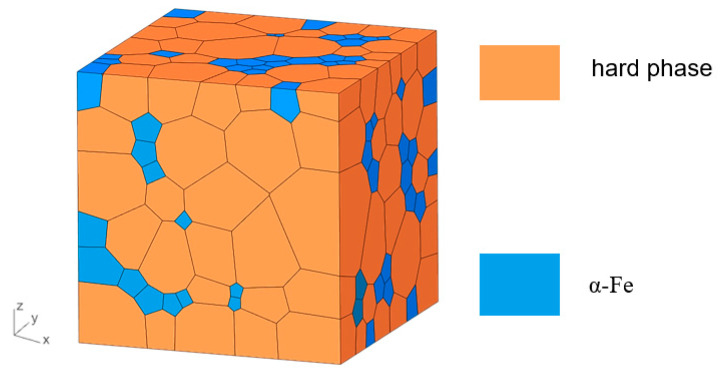
Micromagnetic model of nanocomposite magnet when the grain size of hard phase is 40 nm, the grain size of α-Fe is 10 nm, and the volume fraction of α-Fe is 20%.

Micromagnetism is a theory based on classical field theory and energy minimization. The basic equations of micromagnetism are the energy minimization equation and the Landau–Lifshitz–Gilbert (LLG) equation [9], shown as Equations (1) and (2):

(1) $$E_{t}=E_{ex}+E_{a}+E_{d}+E_{H}=f(M)$$

(2) $$\frac{\delta M}{\delta t}=\alpha M\times H_{eff}+\beta M\times (M\times H_{eff})$$

where ***M*** is the magnetization vector. *E_t_* is the total energy in the magnet. *E_ex_*, *E_a_*, *E_d_*, and *E_H_* represent exchange coupling energy, anisotropic energy, stray energy, and Zeeman energy, respectively. Equation (2) represents the dynamic equation of the magnetic moment, and the effective field in the magnet can be expressed as $H_{eff}=-\frac{1}{M_{s}}\frac{\delta E_{t}}{\delta M}$. *α* and *β* are the rotation coefficient and damping coefficient in the dynamic equation, respectively. In this work, a nanocomposite model based on irregularly shaped grains was established, as shown in Figure 1, where the hard magnetic phase is Ce_2_Fe_14_B or Nd_2_Fe_14_B and the soft magnetic phase is α-Fe.

Hertz [10] and Fischer et al. [11] proposed an equation to calculate the characteristic exchange coupling length *l^ex^* of magnetic materials. The exchange coupling formula of soft magnetic material is *l^ex^ =* (*A*/*K*_1_)^1/2^, and that of the hard magnetic phase is *l^ex^ = π*(*A*/*K*_1_)^1/2^. Therefore, the length of exchange coupling between hard-phase grains and α-Fe grains is obtained from these formulas, as shown in Equations (3)–(6).

(3) $$l_{hh}^{ex}={\pi \left(\frac{A_{h}}{K_{1h}}\right)}^{1/2}$$

(4) $$l_{ss}^{ex}={\left(\frac{A_{s}}{K_{1s}}\right)}^{1/2}$$

(5) $$l_{hs}^{ex}={\pi \left(\frac{\sqrt{A_{h}A_{s}}}{K_{1h}}\right)}^{1/2}$$

(6) $$l_{sh}^{ex}={\left(\frac{\sqrt{A_{h}A_{s}}}{K_{1s}}\right)}^{1/2}$$

In the above equations, *h* and *s* represent the hard magnetic phase and the α-Fe phase, respectively, and $l_{hh}^{ex}$, $l_{ss}^{ex}$, $l_{hs}^{ex}$, and $l_{sh}^{ex}$ represent the exchange coupling length in the hard magnetic phase, in the α-Fe phase, between the hard magnetic and α-Fe phases, and between α-Fe and hard magnetic phases, respectively. $K_{1h}$ and $K_{1s}$ represent the magnetic anisotropy coefficients of the hard phase and the α-Fe phase, respectively. $A_{h}$ and $A_{s}$ represent the exchange interaction coefficients of the hard phase and the α-Fe phase, respectively. The parameters used to calculate the characteristic length exchange coupling can be found in Table 1. Based on Equations (3)–(6), the exchange length of the Ce_2_Fe_14_B/α-Fe nanocomposite system is calculated as $l_{hh}^{ex}\approx 5.6\;nm$, $l_{ss}^{ex}\approx 23.3\;nm,$ $l_{hs}^{ex}\approx 8.5\;nm$, and $l_{sh}^{ex}\approx 15.4\;nm$, and the exchange length of the Nd_2_Fe_14_B/α-Fe nanocomposite system is calculated as $l_{hh}^{ex}\;\approx 4.2\;nm$, $l_{ss}^{ex}\approx 23.3\;nm,$ $l_{hs}^{ex}\approx 5.64\;nm$, and $l_{sh}^{ex}\approx 17.4\;nm$.

The solver of simulations employed in this work is *magpar* [15]. The energy minimization of the system is solved by the conjugate gradient method. The volume fraction of the hard magnetic phase is varied from 40% to 80%. Considering that the superparamagnetic phenomenon will occur when the grain size is too small, the grain sizes of hard magnetic grains and α-Fe grains are varied from 10 nm to 40 nm. Due to the limitation of computing resources, the number of grains in the model is limited to 100 to 400, and the size of the model is 100 nm to 300 nm according to the different sizes and proportions of hard and soft magnetic grains. 

When two hard magnetic grains are in contact with each other, the exchange coupling length between two adjacent grains is the smallest, as shown in Equations (3)–(6). In order to correctly describe the change of magnetic moments in each grain, the size of the mesh in the micromagnetic model must be smaller than the characteristic length of the exchange coupling. In order to make the calculation results more accurate, the size of the finite element mesh is set to 1 nm when dividing the model mesh. In order to represent the isotropic nanocomposite permanent magnet, the orientation of each grain in the model is random. For convenience, we express the volume fraction of α-Fe grains as V_s_, and the sizes of hard magnetic grains and α-Fe grains as D_h_ and D_s_, respectively. Especially, when the sizes of α-Fe grains and hard magnetic grains are the same, they are expressed as D, where D = D_h_ = D_s_.

### 2.2. Experimental Methods

Ce_12_Fe_84+x_B_6_ (x = 2, 6, 14, 26) alloy ingots are prepared by arc melting under a high-purity Ar atmosphere (99.999%) using the raw materials Ce, Fe, and FeB with purities over 99.9%, and the raw materials were purchased from Zhongnuo Advanced Material (Beijing, China) Technology Co., Ltd. (Beijing, China). The alloy ribbons were obtained by melt-spinning at a wheel speed of 35 m/s. In order to obtain nanocomposite structure, as-spun ribbons with amorphous structure were annealed at a temperature of 650 °C for 10 to 20 min. The phase constitution was identified by X-ray diffraction with a Cu-Kα source (XRD, X’ Pert Pro, PANalytical, Malvern, UK). The magnetic properties were measured by a vibrating sample magnetometer in the physical property measurement system (PPMS, Quantum Design, San Diego, CA, USA). 

## 3. Results and Discussion

### 3.1. Simulations for the Nanocomposites with Uniform Grain Size

**Figure 2:**
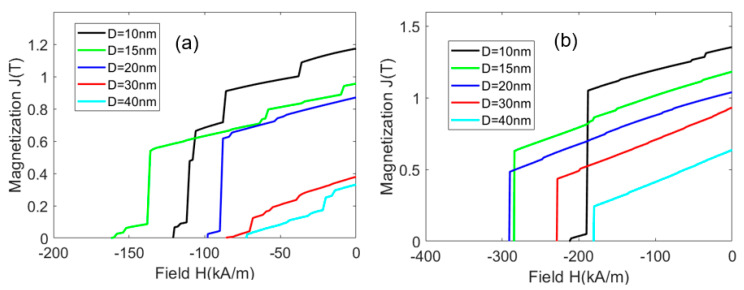
Demagnetization curves of Ce_2_Fe_14_B/α-Fe (**a**) and Nd_2_Fe_14_B/α-Fe (**b**) nanocomposites with different grain sizes D and hard magnetic phase volume fraction V_h_ = 50%.

The demagnetization curves for Ce_2_Fe_14_B/α-Fe and Nd_2_Fe_14_B/α-Fe nanocomposites were first simulated. When the hard magnetic grain and α-Fe have the same size, i.e., D_h_ = D_s_ = D, and the volume fraction of hard grain is 50%, the demagnetization curves are shown in Figure 2a,b for Ce_2_Fe_14_B/α-Fe and Nd_2_Fe_14_B/α-Fe nanocomposites, respectively. The results show that with the increase in grain size, the remanence decreases monotonically and the coercivity first increases and then decreases. 

In the nanocomposite, the magnetic moments of two directly contacted grains at the interface will interact with each other, which prevents the magnetic moments from being oriented in the easy magnetization direction. These magnetic moments continuously change their orientation at the interface from the easy magnetization direction of one grain to the easy magnetization direction of another grain, so that the magnetic moments with disordered orientation tend to be arranged in parallel as much as possible. Since the exchange coupling distance between different grains is constant, with the reduction in grain size, more volume fraction of exchange coupling region exists between the grains, and there are more consistently arranged magnetic moments in the whole magnet in the relaxed state. As a result, the remanence is enhanced. When the volume fraction of hard magnetic grains is 50%, the distribution of hard magnetic grains and α-Fe grains in the whole model is very uniform. Based on Equations (3)–(6), the exchange coupling length between Ce_2_Fe_14_B and α-Fe grains is 15.4 nm, and that between Nd_2_Fe_14_B and α-Fe grains is 17.4 nm. Therefore, when the grain size is larger than 15.4 nm, some α-Fe grains have regions that are not coupled by Ce_2_Fe_14_B grains. Because the anisotropic field of α-Fe is weak, when the grain size of the Ce_2_Fe_14_B/α-Fe model is greater than 15.4 nm, the coercivity of the magnet decreases. Similarly, when the grain size of the Nd_2_Fe_14_B/α-Fe model is greater than 17.4 nm, the coercivity of the magnet decreases. However, the anisotropic field of Nd_2_Fe_14_B is much stronger than that of Ce_2_Fe_14_B; hence, the coercivity of the Nd_2_Fe_14_B/α-Fe model only decreases when the grain size is greater than 20 nm.

**Figure 3:**
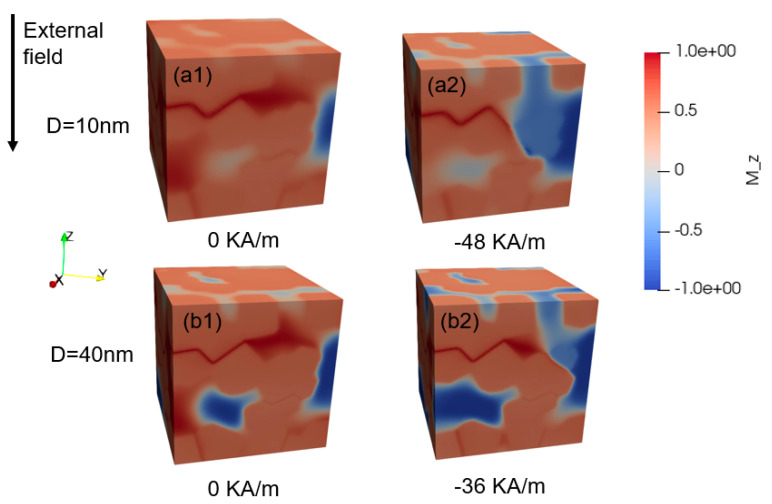
Demagnetization process of the Ce_2_Fe_14_B/α-Fe nanocomposites with D = 10 nm (**a1**,**a2**) and D = 40 nm (**b1**,**b2**). Red regions mean the positive magnetization direction, and blue regions mean the negative magnetization direction.

The Ce_2_Fe_14_B/α-Fe model is used as an example for investigating the demagnetization process of nanocomposite magnets. Figure 3 shows the demagnetization processes of the Ce_2_Fe_14_B/α-Fe model with D = 10 nm (a1, a2) and D = 40 nm (b1, b2). Figure 3(a1,b1) shows the magnetization distribution of these two models in the relaxation state. It can be found that the exchange coupling in the model with a small grain size is stronger, resulting in fewer areas of negative magnetization and a more consistent orientation of the magnetic moments. Figure 3(a2,b2) shows that under the external magnetic field, the demagnetization area of the model with a smaller grain size is more continuous. As we know, after the hard magnetic phase grain size exceeds the exchange length of the soft and hard phases, there will be some areas in the hard grain not subject to the exchange coupling of adjacent grains, and these areas will lag behind other areas during demagnetization.

**Figure 4:**
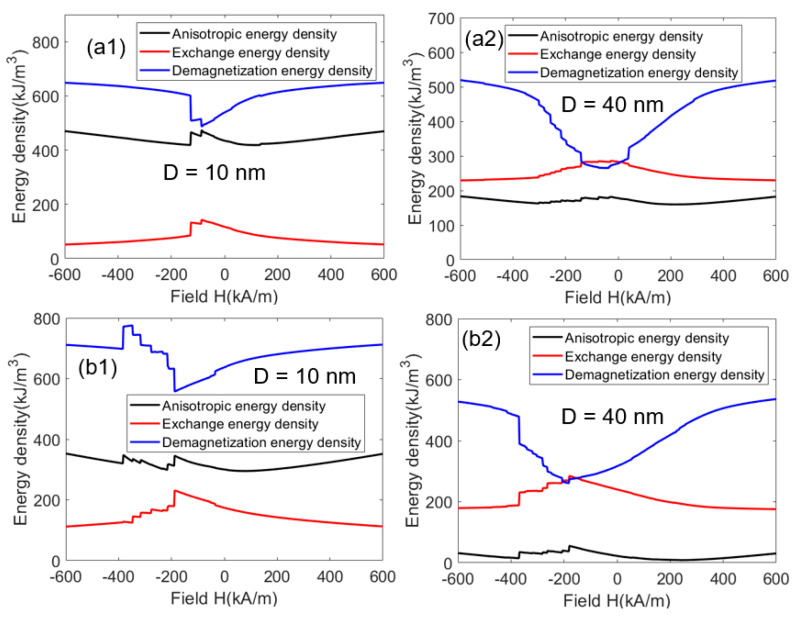
Variations in calculated energy density with external magnetic field for the Ce_2_Fe_14_B/α-Fe (**a1**,**a2**) and Nd_2_Fe_14_B/α-Fe (**b1**,**b2**) nanocomposites with grain size D = 10 nm and D = 40 nm.

Figure 4 shows the variations in anisotropic energy density, exchange energy density, and demagnetization energy density with an external magnetic field for the nanocomposites with grain sizes of 10 nm and 40 nm. Here, the anisotropic energy density is the anisotropic energy divided by the volume of the magnet. Similar definitions are also for exchange energy density and demagnetization energy density. According to Equation (1), when the directions of the magnetic moments and the anisotropic field are parallel or antiparallel, the anisotropy energy is the smallest. The lower the anisotropic energy, the higher the coercivity. In ferromagnetic materials, when two adjacent magnetic moments are parallel to each other, the exchange energy of the magnetic moments is the smallest, but the demagnetization energy is the highest. Figure 4(a1,b1) shows that the exchange energy density is always less than the anisotropic energy density when the grain size is 10 nm, and Figure 4(a2,b2) shows that the exchange energy density is always greater than the anisotropic energy density when the grain size is 40 nm. These results indicate that when the distribution of hard magnetic grains and α-Fe grains is uniform, the smaller grain size can lead to sufficient exchange coupling, which will increase the remanence. At the same time, it will decrease the anisotropy and make the arrangement of magnetic moments inconsistent with the direction of the anisotropic field. The magnetic moments are more likely to be deflected by the external magnetic field, resulting in decreased coercivity. However, in most cases, the demagnetization energy density is much higher than the other two energy densities, indicating that the minimization of demagnetization energy plays a decisive role in the process of minimizing the total energy of the magnet. It can even be said that the energy minimization of a system is the minimization of demagnetization energy. Therefore, as shown in Figure 3, after the grain size increases, the magnetization of some areas will be negative, so as to reduce the demagnetization energy. 

It is also found in Figure 4 that for D = 10 nm, the grain size is smaller than $l_{sh}^{ex}$, and the demagnetization energy is large. Under the external magnetic field, in order to reduce the demagnetization energy, the soft magnetic phase will reverse first, forming a configuration where the magnetic moments of the hard phase and soft phase are anti-parallel. Since the saturation magnetization *M_s_* of Ce_2_Fe_14_B is lower than that of Nd_2_Fe_14_B, the demagnetization energy of Ce_2_Fe_14_B/α-Fe is lower than that of Nd_2_Fe_14_B/α-Fe. Compared with Nd_2_Fe_14_B/α-Fe, the demagnetization energy of Ce_2_Fe_14_B/α-Fe can be minimized by only requiring smaller demagnetization areas, and this can be realized by a relatively low external magnetic field. In addition to the lower anisotropy field of Ce_2_Fe_14_B than Nd_2_Fe_14_B, the lower *M_s_* is also the reason why the coercivity of Ce_2_Fe_14_B/α-Fe is lower than that of Nd_2_Fe_14_B/α-Fe. For D = 40 nm, in the same way, Ce_2_Fe_14_B with lower *M_s_* will lead to lower coercivity of Ce_2_Fe_14_B/α-Fe. When the external magnetic field is 0, the demagnetization energy density of Ce_2_Fe_14_B/α-Fe is smaller than the exchange energy density. However, for Nd_2_Fe_14_B/α-Fe, a larger negative external magnetic field is required. This is mainly because the exchange coupling length of the hard and soft magnetic phases of Ce_2_Fe_14_B/α-Fe is smaller than that of Nd_2_Fe_14_B/α-Fe, and the uncoupled region in Ce_2_Fe_14_B/α-Fe is larger. To minimize the demagnetization energy, the uncoupled region in Ce_2_Fe_14_B/α-Fe is more prone to demagnetization compared to that of Nd_2_Fe_14_B/α-Fe. This is also the reason why the remanence decreases sharply after the grain size of Ce_2_Fe_14_B/α-Fe exceeds $l_{sh}^{ex}$.

### 3.2. Simulations for the Nanocomposites with Different Hard and Soft Magnetic Grain Sizes

**Figure 5:**
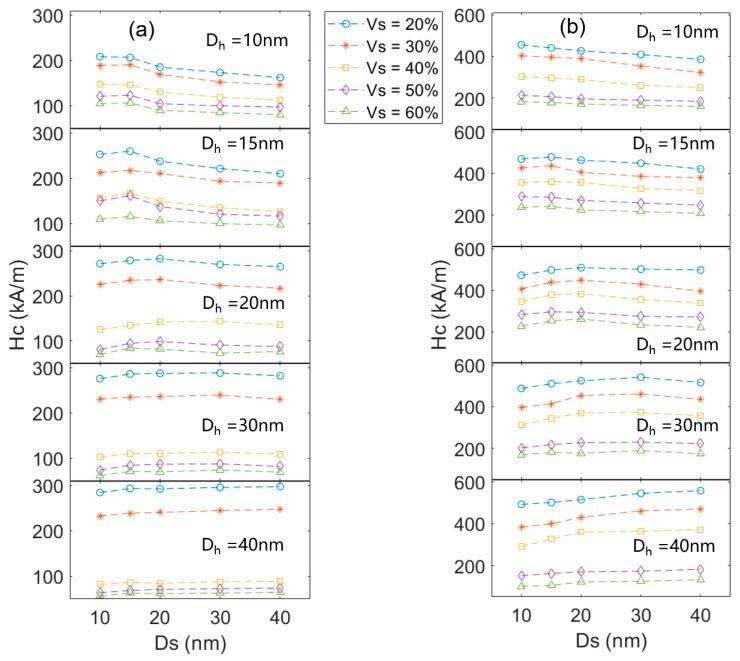
Calculated coercivities of the nanocomposites with different hard magnetic grain and α-Fe grain sizes at different volume fractions. (**a**,**b**) represent the Ce_2_Fe_14_B/α-Fe and Nd_2_Fe_14_B/α-Fe nanocomposites, respectively.

**Figure 6:**
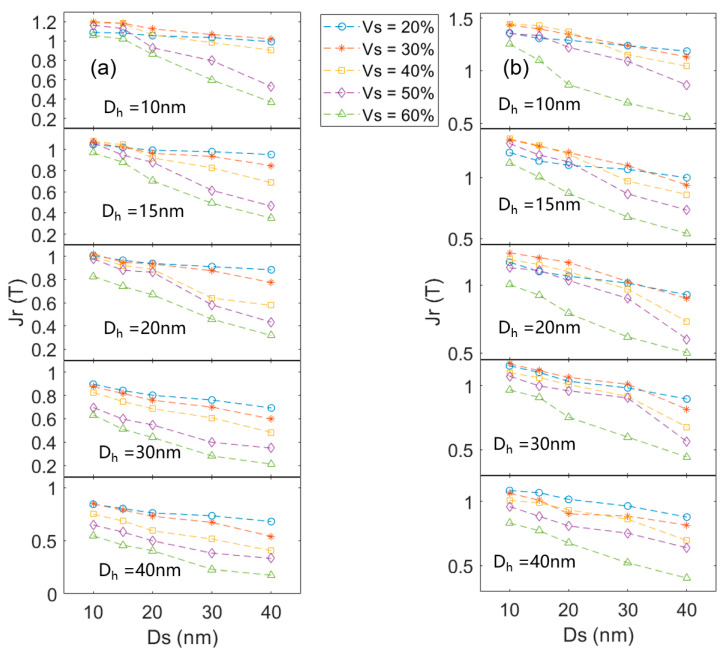
The remanence of the nanocomposites with different hard magnetic grain sizes and α-Fe grain sizes at different volume fractions. (**a**,**b**) represent the Ce_2_Fe_14_B/α-Fe and Nd_2_Fe_14_B/α-Fe nanocomposites, respectively.

In actual nanocomposites, the grain sizes of the hard magnetic phase and the soft magnetic phase are often not the same [16,17,18]. Figure 5 and Figure 6 show the coercivity and remanence of the nanocomposites with different hard magnetic and α-Fe grain sizes at different volume fractions. In general, a smaller grain size leads to higher remanence, and a lower volume fraction of the α-Fe phase results in higher coercivity. 

Figure 5 shows that the coercivity is maximized when the grain sizes of α-Fe and hard magnetic phases are close to each other under the same volume fraction of the α-Fe phase, no matter what the α-Fe fraction is. When the α-Fe grain size is smaller than the hard magnetic grain size, the number of α-Fe grains will increase and the surface area of α-Fe grains will increase. The increased contact area of α-Fe grains and hard magnetic grains will increase the exchange coupling area of hard magnetic grains by α-Fe grains. As a result, the coercivity decreases. When the grain size is smaller than the exchange length of the hard and soft magnetic phases, increasing the grain size of α-Fe results in little change in the coercivity of the composite. Taking the Ce_2_Fe_14_B/α-Fe model as an example, when Ce_2_Fe_14_B grain size is 10 nm, and α-Fe grain size increases from 10 nm to 15 nm, the coercivity is basically unchanged, because α-Fe grain size is within the characteristic exchange length of hard and soft magnetic phases. When the grain size is larger than the characteristic exchange length and the α-Fe grain is larger than the hard grain, there is an area in the α-Fe grain that is not coupled by the hard magnetic grain, which leads to reduced coercivity. 

Figure 5 also shows that when the size of hard magnetic grains is constant, the lower volume fraction of α-Fe grain leads to greater coercivity. This is because the anisotropic field of hard magnetic grains is stronger than that of α-Fe. When the α-Fe phase volume fraction is less than 30%, the larger size of the Ce_2_Fe_14_B grain leads to greater coercivity for the same volume fraction of α-Fe. However, for the Nd_2_Fe_14_B/α-Fe system, this situation occurs when the α-Fe fraction is less than 50%. This is because the exchange coupling length of α-Fe and Nd_2_Fe_14_B is larger, and the exchange coupling area between Nd_2_Fe_14_B and α-Fe is larger. When the size of the Ce_2_Fe_14_B grain reaches 20 nm, with further increasing Ce_2_Fe_14_B grain size, the coercivity does not change significantly. When the α-Fe content is less than 30%, the proportion of the Ce_2_Fe_14_B phase is relatively high, and more Ce_2_Fe_14_B grains will contact each other. According to Equations (3)–(6), the exchange coupling area of the hard magnetic phase subjected to adjacent grains is always small. When the grain size of the hard magnetic phase increases to a certain extent, some hard magnetic phase grains will have areas that are not subject to the exchange coupling of adjacent grains. When the area that is not subject to exchange coupling increases to a certain extent, the anisotropic energy in the hard magnetic phase will be less than the exchange energy. Hence, as the grain size of the hard magnetic phase continues to increase, the coercivity has no significant change. Due to the fact that the exchange coupling length between Nd_2_Fe_14_B grains is always smaller than that of Ce_2_Fe_14_B grains, for the Nd_2_Fe_14_B/α-Fe system, this situation occurs when the Nd_2_Fe_14_B grain size reaches 15 nm. 

However, when the volume fraction of α-Fe grains is greater than 30%, the larger Ce_2_Fe_14_B grain size will result in lower coercivity. This is because, for the high content of α-Fe, the exchange coupling area of α-Fe grains to Ce_2_Fe_14_B grains is large. When the Ce_2_Fe_14_B grain size becomes larger, the number of Ce_2_Fe_14_B grains will decrease, and the surface area of Ce_2_Fe_14_B grains contacting α-Fe grains will decrease. The decreased coupling area will lead to decreased coercivity. For the Nd_2_Fe_14_B/α-Fe model, this situation occurs when the volume fraction of α-Fe grains is greater than 50%. As mentioned above, in addition to the larger exchange length of hard and soft magnetic phases, the higher *M_s_* of Nd_2_Fe_14_B is also a reason. In the nanocomposite system, the α-Fe phase reverses first, while the Nd_2_Fe_14_B has a higher *M_s_*. Lowering the demagnetization energy of Nd_2_Fe_14_B/α-Fe not only requires a larger external magnetic field but also a larger volume of α-Fe phase to reverse. As a result, the Nd_2_Fe_14_B/α-Fe system is able to accommodate a greater volume fraction of the α-Fe phase to maintain high hard magnetic properties. 

Figure 6 shows that when the volume fraction of the hard magnetic phase is constant, the smaller size of Ce_2_Fe_14_B and α-Fe grains lead to higher remanence. The smaller grain size gives a more significant exchange coupling between the grains and lower exchange interaction energy. As a result, the magnetic moments tend to be arranged in parallel, which leads to higher magnetization in the relaxed state. When the size of the α-Fe grain keeps constant, a large hard grain leads to lower remanence. When the size of the hard magnetic grain keeps constant, the larger α-Fe size results in lower remanence. The reasons are rather clear. A larger grain size gives a smaller area of exchange coupling between the soft and hard grains, which decreases the remanence. When the volume fraction of the hard magnetic phase is 60–70% and the sizes of Ce_2_Fe_14_B and α-Fe grains are 10 nm, the maximum remanence can be obtained. Because in this case, all α-Fe grains with a size smaller than the exchange coupling length can be evenly dispersed in the model, the exchange coupling area in the whole magnet is maximized. As a result, the advantage of the high saturation magnetization of the α-Fe phase can be fully utilized.

**Figure 7:**
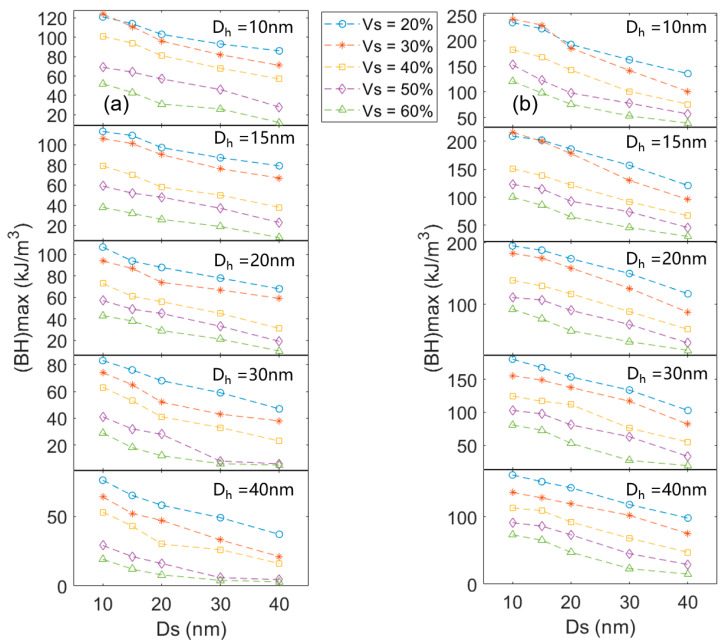
The maximum magnetic energy product of the nanocomposites with different hard magnetic grain sizes and α-Fe grain sizes at different volume fractions. (**a**,**b**) represent the Ce_2_Fe_14_B/α-Fe and Nd_2_Fe_14_B/α-Fe nanocomposites, respectively.

The maximum magnetic energy product is an important index to evaluate the properties of permanent magnet materials. Figure 7 shows the maximum magnetic energy product (*BH*)*_max_* of the nanocomposites with different grain sizes and different α-Fe volume fractions. The larger grain size leads to the lower (*BH*)*_max_*. For nanocomposites, when the value of coercivity is not very different, the value of (*BH*)*_max_* is positively correlated with the remanence [8]. It is clear that the remanence of nanocomposite reaches its maximum when the grain size is 10 nm and the volume fraction of the hard magnetic phase is 70%. Hence, when the volume fraction of the hard magnetic grain is 70% and the size of the hard magnetic grain and the α-Fe grain is 10 nm, the (*BH*)*_max_* value is maximized. The (*BH*)*_max_* values of 127.65 kJ/m^3^ and 247.58 kJ/m^3^ are obtained in the Ce_2_Fe_14_B/α-Fe and Nd_2_Fe_14_B/α-Fe composites, respectively. In addition, compared to the volume fraction of the Ce_2_Fe_14_B phase, the grain size has a greater effect on the (*BH*)*_max_*. The main reason is that the influence of grain size on the remanence is greater than that of the volume fraction of the hard magnetic phase.

### 3.3. Experimentation and Discussion

**Figure 8:**
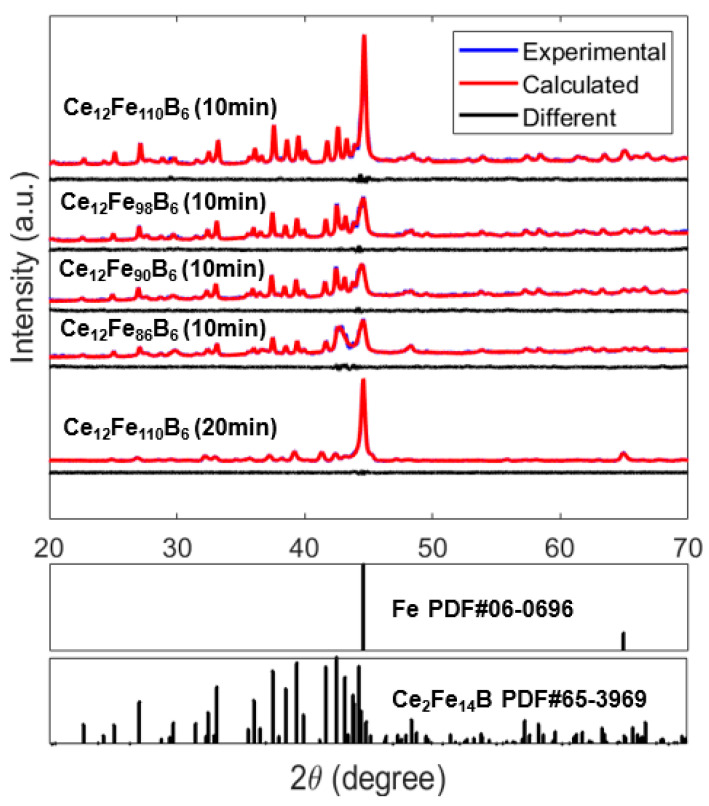
XRD patterns for melt Ce_12_Fe_84+x_B_6_ (x = 2, 6, 14, 26) ribbons obtained for circumferential wheel velocities of 35 m/s, after annealing at 650 °C for different times.

To verify the reliability of the simulation, we prepared Ce_2_Fe_14_B/α-Fe nanocomposite alloys with various hard magnetic phase and soft magnetic phase ratios by melt-spinning. Figure 8 shows the XRD patterns for Ce_12_Fe_84+x_B_6_ (x = 2, 6, 14, and 26) ribbons obtained for a circumferential wheel velocity of 35 m/s after annealing at 650 °C for different times. All Ce_12_Fe_84+x_B_6_ (x = 2, 6, 14, 26) alloys are mainly composed of Ce_2_Fe_14_B and α-Fe phases. Based on the Rietveld analysis of XRD data, the lattice parameters of Ce_2_Fe_14_B have been estimated as a = 0.876 and c = 1.211 nm. According to Scherrer’s formula, after 10 min of heat treatment at 650 °C, the grain sizes of Ce_2_Fe_14_B and α-Fe are 32.6 nm and 14.7 nm, respectively. After 20 min of heat treatment, the grain sizes of Ce_2_Fe_14_B and α-Fe are 42.8 nm and 27.2 nm, respectively. As the content of Fe increases, the characteristic peak of the α-Fe phase increases, indicating an increasing soft magnetic phase. The results verify that Ce_12_Fe_84+x_B_6_ (x = 2, 6, 14, 26) ribbons can form a structure of nanocomposite.

**Figure 9:**
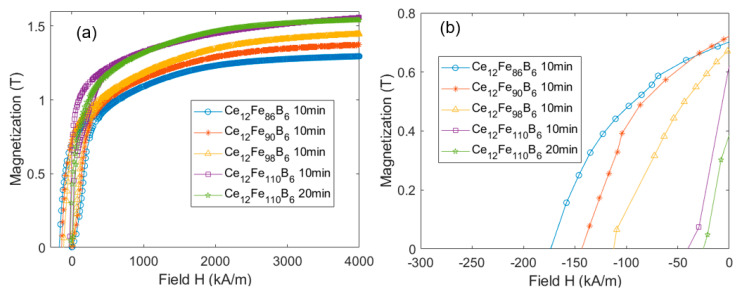
Magnetization (**a**) and demagnetization (**b**) curves of Ce_12_Fe_84+x_B_6_ (x = 2, 6, 14, 26) alloys at different annealing times at 650 °C.

Figure 9 shows the magnetization and demagnetization curves of Ce_12_Fe_84+x_B_6_ (x = 2, 6, 14, 26) ribbons annealed at 650 °C for different times. Their magnetic properties are listed in Table 2. Ce_12_Fe_86_B_6_ and Ce_12_Fe_90_B_6_ exhibit remanence enhancement effects with a remanence ratio of *J_r_*/*J_s_* > 0.5. With the increase in Fe content, the *M_s_* of the alloy increases, and the coercivity decreases. At the same time, *J_r_*/*J_s_* decreases. Since the main peaks in Figure 8 are Ce_2_Fe_14_B and α-Fe, and the other phases can be ignored, the volume fraction of Ce_2_Fe_14_B in the nanocomposite magnet can be calculated according to the *M_s_* of Ce_2_Fe_14_B, α-Fe, and the nanocomposite. Based on the *M_s_* of Ce_12_Fe_84+x_B_6_ (x = 2, 6, 14, 26), the volume fractions of Ce_2_Fe_14_B can be calculated as about 85%, 80%, 70%, and 60%, respectively. The coercivity of Ce_12_Fe_86_B_6_, Ce_12_Fe_90_B_6_, and Ce_12_Fe_98_B_6_ decreases linearly, and the remanence changes slightly. Compared to Ce_12_Fe_98_B_6_, the coercivity of Ce_12_Fe_110_B_6_ decreases significantly, and the remanence decreases slightly. The simulation results in Figure 5a show that when the Ce_2_Fe_14_B grain size is greater than 20 nm and the volume fraction of Ce_2_Fe_14_B is less than 70%, the coercivity of nanocomposites will decrease significantly. The simulation results in Figure 6a also indicate that when the volume fraction of Ce_2_Fe_14_B is greater than 70% and the grain size of α-Fe is less than 30 nm, the remanence of nanocomposites does not change much. When the volume fraction of Ce_2_Fe_14_B is 60%, the remanence will slightly decrease. Thus, the experimental results are in very good agreement with the simulation ones.

As is well known, a longer heat treatment time would result in a larger grain size of the melt-spun ribbons. The annealing time of Ce_12_Fe_110_B_6_ (20 min) is twice that of Ce_12_Fe_110_B_6_ (10 min), and the grain size of Ce_12_Fe_110_B_6_ (20 min) is larger than that of Ce_12_Fe_110_B_6_ (10 min). Compared to Ce_12_Fe_110_B_6_ (10 min), the coercivity of Ce_12_Fe_110_B_6_ (20 min) does not decrease much, and the remanence of Ce_12_Fe_110_B_6_ (20 min) decreases significantly, resulting in a significant reduction in the (*BH*)*_max_*. Figure 5a and Figure 6a show that when the volume fraction of Ce_2_Fe_14_B is 60%, with the increases in the grain sizes of Ce_2_Fe_14_B and α-Fe from 30 nm and 15 nm to 40 nm and 30 nm, respectively, the coercivity of nanocomposites does not decrease much with the increased grain size, but the remanence decreases significantly. Figure 5a, Figure 6a and Figure 9 show that the coercivity of nanocomposites will decrease with the decrease in the volume fraction of Ce_2_Fe_14_B. When the volume fraction of Ce_2_Fe_14_B is less than 70%, the coercivity will decrease sharply, and increasing the grain size has little effect on the coercivity, but the remanence will decrease significantly. Hence, the experimental results are also in good agreement with the simulation results.

From the experimental results, when the volume fraction of α-Fe is greater than 30%, the coercivity of the nanocomposite drops significantly, which not only indicates that the exchange coupling length of the hard and soft magnetic phases of Ce_2_Fe_14_B/α-Fe is indeed smaller than that of Nd_2_Fe_14_B/α-Fe, but also shows that this is caused by the lower saturation magnetization of the Ce_2_Fe_14_B phase. When the grain size of nanocomposite magnets is increased, the uncoupled area increases, and the demagnetization energy of nanocomposites decreases. As a result, both the coercivity and remanence decrease. The present results indicate that the influence of grain size on magnetic properties seems to be greater than that of the volume fraction of hard magnetic phases. Overall, the experimental results are consistent with those of the micromagnetic simulation mentioned above.

## 4. Conclusions

In this work, the magnetic properties of isotropic Ce_2_Fe_14_B/α-Fe and Nd_2_Fe_14_B/α-Fe nanocomposites with different volume fractions of hard magnetic phase and different grain sizes were systematically simulated. When the grain size of both the hard and soft magnetic phases is 10 nm and the volume fraction of the hard magnetic phase is 70%, the highest maximum magnetic energy product can be obtained. When the soft magnetic phase in Ce_2_Fe_14_B/α-Fe nanocomposite exceeds 30%, its magnetic properties will significantly decrease, while this situation only occurs when the volume fraction of the soft magnetic phase in Nd_2_Fe_14_B/α-Fe exceeds 40%. The reason is not only because of the low anisotropic field and the smaller exchange coupling length between the soft magnetic phase and the hard magnetic phase, but also because the lower saturation magnetization of the hard phase will minimize the demagnetization energy of nanocomposites under a lower negative external magnetic field. The experimental results also indicate that the volume fraction of the hard magnetic phase should not be less than 70%, and the grain size has a greater impact on magnetic properties compared to the volume fraction of the hard magnetic phase. The Ce_2_Fe_14_B/α-Fe nanocomposite alloys with various hard/soft magnetic phase ratios and grain sizes were prepared by melt-spinning and heat treatment. The experimental results of magnetic properties on the Ce_2_Fe_14_B/α-Fe system are consistent with the micromagnetic simulation results. The hard magnetic phase with larger saturation magnetization is beneficial to the preparation of high-performance nanocomposite magnets.

## Figures and Tables

**Table 1:** Magnetic parameters used in the simulation [12,13,14].

	*K*_1_ (MJ/m^3^)	*J_s_* (T)	*A* (pJ/m)
Ce_2_Fe_14_B phase	1.5	1.17	4.8
α-Fe phase	0.048	2.15	25
Nd_2_Fe_14_B phase	4.5	1.61	7.7

**Table 2:** The magnetic properties of melt-spun Ce_12_Fe_84+x_B_6_ (x = 2, 6, 14, 26) alloys under different annealing treatments.

Alloy	Annealing	*H_c_* (kA/m)	*J_r_* (T)	*J_s_* (T)	(*BH*)*_max_* (kJ/m^3^)
Ce_12_Fe_86_B_6_	650 °C 10 min	172	0.70	1.30	37.65
Ce_12_Fe_90_B_6_	650 °C 10 min	141	0.71	1.37	32.88
Ce_12_Fe_98_B_6_	650 °C 10 min	112	0.67	1.44	20.23
Ce_12_Fe_110_B_6_	650 °C 10 min	41	0.62	1.54	5.74
Ce_12_Fe_110_B_6_	650 °C 20 min	24	0.38	1.55	2.08

## Data Availability

Seek out the author to get the raw data.

## References

[1] Withanawasam L., Hadjipanayis G., Krause R.F. (1994). Enhanced remanence in isotropic Fe-rich melt-spun Nd-Fe-B ribbons. J. Appl. Phys..

[2] Withanawasam L., Zheng Y.H., Hadjipanayis G.C., Krause R.F. (1995). Nanocomposite RE_2_Fe_14_B/α-Fe and Sm_2_(Fe-Ga)_17_C_x_/α-Fe magnets. Scr. Metall. Mater..

[3] Coehoorn R., de Mooij D.B., de Waard C. (1989). Meltspun permanent magnet materials containing Fe_3_B as the main phase. J. Magn. Magn. Mater..

[4] Kneller E.F., Hawig R. (1991). The exchange-spring magnet: A new material principle for permanent magnets. IEEE Trans. Magn..

[5] Coey J.M.D., Skomski R. (1993). Giant energy product in nanostructured two-phase magnets. Phys. Rev. B.

[6] Ryo H., Kim K., Kim Y. (2019). An analytic study on coercivity mechanism of exchange coupled Nd_2_Fe_14_B/α-Fe nanocomposite magnets. J. Magn. Magn. Mater..

[7] Wysocki A.L., Antropov V.P. (2017). Micromagnetic simulations with periodic boundary conditions: Hard-soft nanocomposites. J. Magn. Magn. Mater..

[8] Kim C.S., Zha L., Li M.N., Yang W.Y., Han J.Z., Liu S.Q., Du H.L., Wang C.S., Yang J.B. (2021). Micromagnetic simulation for optimizing nanocomposite Nd_2_Fe_14_B/α-Fe permanent magnets by changing grain size and volume fraction. J. Magn. Magn. Mater..

[9] Aharoni A. (2000). An Introduction to the Theory of Ferromagnetism.

[10] Herzer G. (1990). Grain size dependence of coercivity and permeability in nanocrystalline ferromagnets. IEEE Trans. Magn..

[11] Fischer R., Schrefl T., Kronmüller H., Fidler J. (1996). Grain-size dependence of remanence and coercive field of isotropic nanocrystalline composite permanent magnets. J. Magn. Magn. Mater..

[12] Hirosawa S., Matsuura Y., Yamamoto H., Fujimura S., Sagawa M., Yamauchi H. (1986). Magnetization and magnetic anisotropy of Re_2_Fe_14_B measured on single crystals. J. Appl. Phys..

[13] Herbst J.F. (1991). Re_2_Fe_14_B materials: Intrinsic properties and technological aspects. Rev. Mod. Phys..

[14] Li W., Zhao L., Liu Z. (2019). Micromagnetic simulation for the effects of core-shell distributions of RE on the magnetic properties of dual-main-phase Nd-Fe-B based magnets. J. Magn. Magn. Mater..

[15] Scholz W., Fidler J., Schrefl T., Suess D., Dittrich R., Forster H., Tsiantos V. (2003). Scalable parallel micromagnetic solvers for magnetic nanostructures. Comput. Mater. Sci..

[16] Tao S., Ahmad Z., Zhang P., Zheng X., Zhang S. (2023). Magnetic and structural properties of nanocomposite permanent magnet produced from crystallization of Pr_4_Fe_67_Nb_4_Cu_2_Zr_1_B_22_ alloy. J. Magn. Magn. Mater..

[17] Li Y., Yu N., Wu Q., Pan M., Ge H., Zhu M., Li W. (2023). Novel method for preparation of high-performance SmFe_12_/α-Fe nanocomposite magnets by rapid thermal processing. J. Alloys Compd..

[18] Li H., Fu K., Qian E., Bu X., Li M., Yang A., Zhang H., Xia A. (2022). Study of the role of P doping on microstructure and magnetic properties of Nd_2_Fe_14_B/α-Fe nanocomposite magnets. J. Alloys Compd..

